# Plate Work

**Published:** 1906-06

**Authors:** J. T. J. Watson

**Affiliations:** Lapine.


### PLATE WORK.

By J. T. J. Watson, D. D. S., Lapine.

Alabama Dental Association.

The apostle Peter said he wrote certain things, not because the people to whom he wrote did not know them, but to stir up their pure minds by way of remembrance. So I will be in good company if I stir the minds of the dentists present by putting them in remembrance of things they already know, for certainly no branch of dental science or art has suffered so much from negligence of dentists as has prosthetic dentistry. It is a great misfortune for a person to lose the natural organs of mastication and articulation; and when this mishap does befall a person, he ought to have the very best substitute that human skill can construct. With the best efforts of the dental profession under present environments, the name of those who have lost some or all of the natural teeth must necessarily be legion; hence the happiness, the health, and the longevity of a large part of the human family are, at least to a great extent, at the disposal of the dental profession; and for this reason no dentist ought to be satisfied with himself unless he is able to stand in the front rank of his profession, nor be satisfied with any special service rendered unless it measures up with the possibilities of human ingenuity. With these facts before us, which are known and recognized by all, is it not surprising to see a dentist of high aspirations take an impression with beeswax? Yet this is sometimes done, while all know the liability of wax to drag while taking out of the mouth.

Then I ask: Why do any who regard their obligations and reputation as dentists ever use beeswax for an impression? As the impression is the thing that determines the fit or the misfit of the plate, it is all-important to get an absolutely perfect one. Nothing else known to me is so well suited for taking a correct impression as plaster of Paris. I admit it is a little more unpleasant to both the dentist and the patient; but what is this little unpleasantness compared with the years of discomfort that must follow as a result of a poor impression? I confess that in partial cases it is my custom to use modeling compound for the impression because of the possible difficulty of dislodging a plaster impression; still a "plaster impression may be removed in such cases without difficulty by putting a collar of wax around each tooth to be included in the impression."

For a correct plaster impression, especially in the case of a deep arch, it is a good idea to take a wax impression first; then trim out a portion of the wax in the center, leaving a perfect border of the wax impression. Place plaster in this and take impression; thus we will have but little trouble in getting a correct impression of the highest arch.

It is well known that a lower plate usually gives no little trouble to the wearer while getting used to it, and that even after long use it is a poor substitute for the natural teeth; therefore, when practical, it is best to save one natural bicuspid on each side to anchor to, somewhat after the Marshall method. I hardly deem it necessary to describe this method, for I suppose all understand it. The modification I use, however, is simply an ordinary gold crown on one tooth on each side, a bicuspid preferable, for which a gold band is made to fit accurately, and yet so it will slip on and off readily. This band is attached to the plate in vulcanizing, so that when the plate is in place, the bands are around the gold crowns, thus holding the plate securely in place. The Marshall method of anchoring a lower plate is, I think, some eighteen or twenty years old, possibly more. The telescope band is as good, and, in some respects, better than the telescope crown, and I have seen nothing else to equal it. When properly done, it is, indeed, the next thing to natural teeth. When none of the teeth can be saved, we are at a disadvantage; still, patients do not always get a conscientious effort on the part of the dentist. When the lower jaw is entirely edentulous, there is sure to be more or less lateral movement of the plate, which is irritating to the gums at first, so that the wearing of the plate will be quite a trying ordeal.

Now I suggest, in case of a rubber plate, we use flexible or palate rubber as a kind of cushion or pad, which will make the plate stick to the gums much better, will be less irritating to the gums, and will be much more comfortable and satisfactory to the patient. I will give the method of constructing it, which I get from what used to be a "dental secret." Have the base plate so made that there will be but very little to trim or file from the edge of the plate. Then invest so that the plaster in the lower part of the flask just comes flush with the edge of the wax, so that there will be no small undercut to break away when flask is separated. After you have packed with the required amount of rubber, and before closing the flask, cut half a sheet of starched muslin (that which comes between the rubber sheets), dampen it in warm water, and place between the upper and lower parts of the flask. After closing in the usual manner, separate and remove the muslin. You will find that you have a nice, clean form of your rubber plate, free from plaster or other deleterious matter. Then cut small strips from a sheet of "Dougherty's Flexible Rubber" and spread carefully and smoothly over the rubber form to within one-eighth of an inch of the edge all around. Then close your flask and vulcanize in the usual way. When vulcanized, you will find that you have a nice, soft, flexible bearing to rest on the tissue that will adhere better than an air-chambered plate. This applies to either upper or lower plate, but is more needful for a lower one, from the fact that without it the lower one is more troublesome than the upper one.

One other thing to which I will call your attention, and that is the selection of teeth for any given case. It is important to select teeth that will properly articulate their antagonists. The cusps, or prominences, on the grinding surface are all right and serve a good purpose when properly articulated; but when they cannot be articulated so that prominent points in one arch will meet depression in the other, they serve to discharge the plates, and are, therefore, annoying and a disadvantage. Rather than have them thus, it is better to grind off the cusps. While they will not masticate food so well without cusps as with them when properly articulated, they will stay in place better and be more satisfactory ground off than to be allowed to remain and not properly antagonize the opposing teeth.

